# Supplementary material for: Microcirculation-Promoting Effect of Escin on Cutaneous Tissue via Gsk3β Down-Regulation
Source: Curr Issues Mol Biol. 2025 Oct 14;47(10):840. doi: 10.3390/cimb47100840 (PMC12563160; doi:10.3390/cimb47100840)
Supplement: Supplementary file 1 [file cimb-47-00840-s001.zip › cimb-3844719-supplementary.pdf]

Figure S1. The ratio of p-Gsk3β (Ser9) and total Gsk3β with escin treatment in cultured HUVECs

The levels of phospho-Gsk3β (Ser 9 site), Gsk3β were further investigated by Western blotting with escin. The ratio of phospho-Gsk3β (Ser 9 site), Gsk3β was calculated. Wnt3a were used as positive control. N.T, non-treated control. N.S, Not Significant. The data represent the means of five independent samples.

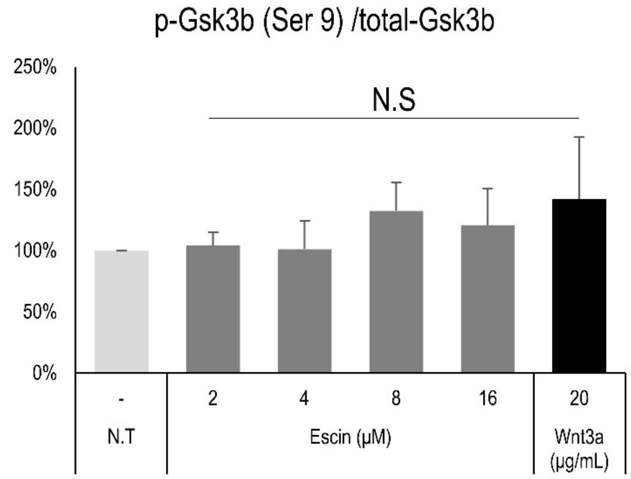

Table S1. Analysis of correlation genes in silico with activated genes in vitro (HUVEC) by escin

# node1	# node2	Combined score
CTNNB1	AKT1	0.999
CTNNB1	GSK3B	0.999
CTNNB1	CREBBP	0.999
CTNNB1	GSK3A	0.988
CTNNB1	FER	0.977
CTNNB1	RPS6KB1	0.684
CTNNB1	RPS6KA4	0.55
CTNNB1	LCK	0.407
JUN	CREBBP	0.999
JUN	MAPK8	0.999
JUN	GSK3B	0.999
JUN	CTNNB1	0.992
JUN	MAPK7	0.982
JUN	AKT1	0.977
JUN	MAP2K3	0.78
JUN	MTOR	0.775
JUN	RPS6KB1	0.652
JUN	LCK	0.643
JUN	EIF4EBP1	0.472
JUN	GSK3A	0.456
JUN	RPS6KA4	0.452
RICTOR	AKT1	0.999
RICTOR	EIF4EBP1	0.999
RICTOR	RPS6KB1	0.999
RICTOR	MAPKAP1	0.999
RICTOR	RPTOR	0.998
RICTOR	GSK3B	0.929
RICTOR	RPS6KA4	0.76
RICTOR	GSK3A	0.625
RICTOR	MAPK7	0.436

Table S2. Reverse screening of escin to target proteins

Target	Common name	Probability
Proto-oncogene c-JUN	JUN	0.042
Protein kinase C alpha	PRKCA	0.042
MAP kinase p38 alpha	MAPK14	0.042
Signal transducer and activator of transcription 3	STAT3	0.042
Tyrosine-protein kinase JAK3	JAK3	0.042
Apoptosis regulator Bcl-X	BCL2L1	0.042
Tyrosine-protein kinase SYK	SYK	0.042
MAP kinase ERK2	MAPK1	0.042
Transient receptor potential cation channel subfamily V member 4	TRPV4	0.042
Apoptosis regulator Bcl-2	BCL2	0.042
Tyrosine-protein kinase JAK1	JAK1	0.042

Table S3. Statistical analysis for baseline level of blood flow evaluation

		**Group 1**	**Group 2**	**Group 3**	**Group 4**
Treatment		Vehicle<br>(Non-treated Control)	Adenosine 0.75%	Adenosine 0.75%<br>+ Escin 0.2%	Adenosine 0.75%<br>+ Escin 0.5%
Number		n = 8	n = 8	n = 7	n = 8
Baseline (A.U)		55.5	45.6	52.9	58.1
Standard Deviation		14.1	13.8	18.9	15.5
One way ANOVA test (Baseline)		p = 0.4850			
Tukey's multiple comparisons test (p - value)	Group 1	-	0.929	0.832	0.990
	Group 2	0.929	-	0.450	0.989
	Group 3	0.832	0.450	-	0.639
	Group 4	0.990	0.989	0.639	-
